# Ancestral exposure to stress epigenetically programs preterm birth risk and adverse maternal and newborn outcomes

**DOI:** 10.1186/s12916-014-0121-6

**Published:** 2014-08-07

**Authors:** Youli Yao, Alexandra M Robinson, Fabiola CR Zucchi, Jerrah C Robbins, Olena Babenko, Olga Kovalchuk, Igor Kovalchuk, David M Olson, Gerlinde AS Metz

**Affiliations:** Canadian Centre for Behavioural Neuroscience, Department of Neuroscience, University of Lethbridge, 4401 University Drive, Lethbridge, AB T1K3M4 Canada; Department of Biological Sciences, University of Lethbridge, 4401 University Drive, Lethbridge, AB T1K3M4 Canada; Departments of Obstetrics & Gynecology, Pediatrics and Physiology, University of Alberta, 227 HMRC, Edmonton, AB T6G2S2 Canada

**Keywords:** Preterm birth, maternal stress, prenatal stress, transgenerational inheritance, microRNA, epigenetic regulation, gestation, maternal health, behavioural development, perinatal programming, pregnancy

## Abstract

**Background:**

Chronic stress is considered to be one of many causes of human preterm birth (PTB), but no direct evidence has yet been provided. Here we show in rats that stress across generations has downstream effects on endocrine, metabolic and behavioural manifestations of PTB possibly via microRNA (miRNA) regulation.

**Methods:**

Pregnant dams of the parental generation were exposed to stress from gestational days 12 to 18. Their pregnant daughters (F1) and grand-daughters (F2) either were stressed or remained as non-stressed controls. Gestational length, maternal gestational weight gain, blood glucose and plasma corticosterone levels, litter size and offspring weight gain from postnatal days 1 to 30 were recorded in each generation, including F3. Maternal behaviours were analysed for the first hour after completed parturition, and offspring sensorimotor development was recorded on postnatal day (P) 7. F0 through F2 maternal brain frontal cortex, uterus and placenta miRNA and gene expression patterns were used to identify stress-induced epigenetic regulatory pathways of maternal behaviour and pregnancy maintenance.

**Results:**

Progressively up to the F2 generation, stress gradually reduced gestational length, maternal weight gain and behavioural activity, and increased blood glucose levels. Reduced offspring growth and delayed behavioural development in the stress cohort was recognizable as early as P7, with the greatest effect in the F3 offspring of transgenerationally stressed mothers. Furthermore, stress altered miRNA expression patterns in the brain and uterus of F2 mothers, including the miR-200 family, which regulates pathways related to brain plasticity and parturition, respectively. Main miR-200 family target genes in the uterus, *Stat5b*, *Zeb1* and *Zeb2*, were downregulated by multigenerational stress in the F1 generation. *Zeb2* was also reduced in the stressed F2 generation, suggesting a causal mechanism for disturbed pregnancy maintenance. Additionally, stress increased placental miR-181a, a marker of human PTB.

**Conclusions:**

The findings indicate that a family history of stress may program central and peripheral pathways regulating gestational length and maternal and newborn health outcomes in the maternal lineage. This new paradigm may model the origin of many human PTB causes.

**Electronic supplementary material:**

The online version of this article (doi:10.1186/s12916-014-0121-6) contains supplementary material, which is available to authorized users.

## Background

Preterm birth (PTB), which is associated with an intrauterine pro-inflammatory state, represents the leading cause of neonatal morbidity and mortality and one of the most critical factors for disease in later life. For example, infants born preterm, that is, born before 37 completed weeks of pregnancy, are at greater risk for mortality, developmental delay and health conditions than infants born at term [1]. In spite of focused research efforts considering the drastic impact of PTB on health outcomes, in more than 50% of cases the causes of PTB remain unknown.

It has been difficult to demonstrate a clear causal relationship in humans [2], although PTB has been recognized as a consequence of severe maternal distress during pregnancy [3,4] or due to preconceptional factors [5]. Earlier reports suggested that adverse perinatal programming by stress may increase the risk of PTB and low birth weight [3,4,6,7]. Cumulative effects of stress seem to be of particular importance to PTB risk [6], which may include repeated exposure to stress across generations. Notably, elevated PTB risk was noted to propagate through generations [8], suggesting that factors determining PTB risk factors may be passed on to the offspring through the maternal lineage. Recent studies focusing on transmission through the male germ line in rodents have suggested that altered stress responses and associated emotional traits are linked to ancestral exposure to environmental toxins [9] and stressful experiences [10–12]. Furthermore, prenatal exposure to endocrine disruptors in female rats [13,14] or to maternal undernutrition in humans [15] have been associated with increased metabolic and endocrine disease risk in the offspring.

The molecular mechanisms leading to stress-induced pathologies in the maternal lineage occur through two different mechanisms. One mechanism occurs through direct exposure of fetal somatic cells in the female F1 and F2 generations [16,17]. Alternatively, if phenotypic changes persist in the non-exposed F3 generation, truly transgenerational mechanisms involve changes in the germline that involve epigenetic mechanisms [16,17]. Possible mechanisms of transgenerational transmission may be linked to a stress-associated epigenotype involving microRNAs (miRNAs) that are replicated in subsequent generations. MicroRNAs (miRNAs) are reasonable candidates for such a role since they are differentially regulated by progesterone during myometrial quiescence and initiation of parturition [18,19].

Here, we proposed that maternal stress or the cumulative effects of recurrent stress influence PTB risk and poor health outcomes across three generations. Using rats, we show that PTB risk, metabolic, endocrine and behavioural outcomes are affected by a single exposure to prenatal stress in one generation. In addition, the findings indicate that recurrent prenatal stress across multiple generations amplifies hypothalamic-pituitary-adrenal (HPA) axis responses to exacerbate variations in gestational lengths and adverse outcomes. We also show that stress-modulated gestational length is accompanied by miRNA expression changes and altered target gene pathways in somatic cells in F1 and F2 generations. Our data suggest that epigenomic programming of PTB risk factors may be an important mechanism involved in adverse pregnancy outcomes and altered maternal and offspring behaviours.

## Methods

### Animals

Four-hundred-and-eight Long-Evans hooded rats (*Rattus norvegicus*), bred and raised at the local vivarium, were used. Nulliparous, pair-housed female rats between the age of 100 and 160 days underwent timed pregnancy by being individually paired with a male for one hour per day until mating occurred. Rats were weighed prior to pregnancy and gestational weight was recorded daily. Pregnancy of the rats was confirmed by steady weight gain. Pregnant rats were housed individually from gestational day (GD) 19 until delivery. The hours of gestational length were monitored by an infrared security system (CCTV Cameras, Panasonic, Newark, NJ, USA). Pups remained with the dams until weaning on postnatal day (P) 21.

The rats were housed under a 12-hour light/day cycle with lights on at 7:30 AM. All procedures were performed in accordance with the guidelines of the Canadian Council for Animal Care and approved by the local Animal Welfare Committee.

### Experimental design

**Figure 1:**
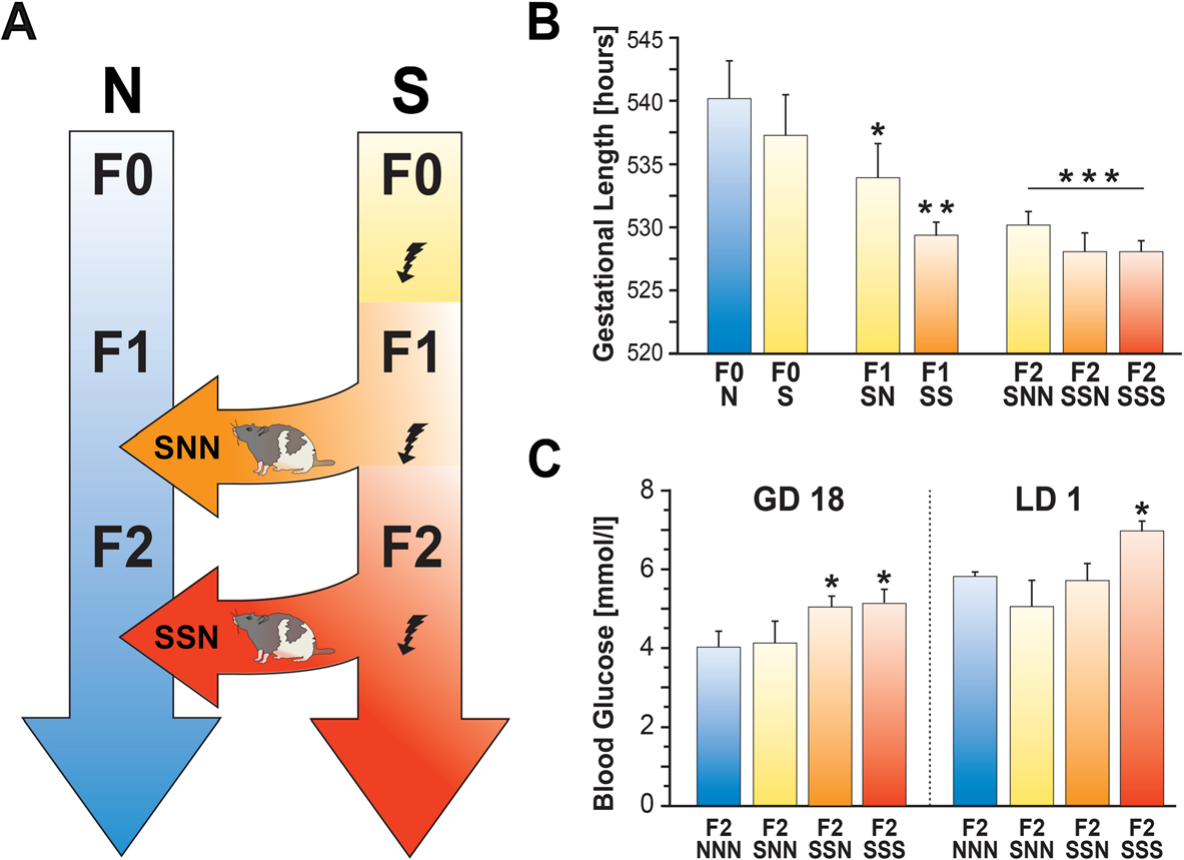
**Prenatal, but not gestational stress, hastens parturition and elevates blood glucose levels. (A)** Flow chart illustrating the experimental design that tested three generations (F0 through F2) of rats and the F3 offspring in which stress occurred only in the parental generation (S, SN, SNN), across multiple generations (S, SS, SSN) or in each generation (S, SS, SSS). Generations (F0 through F2) of non-stressed rats (N, NN, NNN) served as control. Arrows indicate the transfer of F1 and F2 rats from the stressed breeding line to the non-stress condition, generating the SNN and SSN lines, respectively. **(B)** Gestational length recordings showed that gestational stress in the parental generation F0 had no effect on pregnancy duration, while prenatal stress reduced gestational length in subsequent generations. Recurrent stress during pregnancy had additive effects on gestational length. **(C)** Ancestral stress elevated gestational blood glucose levels in animals exposed to multiple generations of stress (F2-SSN or F2-SSS) on gestational day (GD) 18. Elevations persisted to lactational day (LD) 1 in stressed animals whose mothers and grandmothers were also stressed (F2-SSS). S refers to stress, N refers to non-stress control conditions. Asterisks indicate significances: *P <0.05; **P <0.01; ***P<0.001, compared to non-stress controls.

Four successive generations of timed-pregnant female rats (n = 56) were bred under standardized conditions and split by treatment in each generation (see Figure 1A). Parental female rats (F0) were stressed during late gestation (S; n = 10). Their pregnant F1 daughters were split into either stressed (SS; n = 7) or non-stressed groups (SN; n = 5). Their pregnant F2 granddaughters were again either stressed (SSS to represent cumulative effects of stress; n = 9) or not stressed (SNN; n = 10, SSN; n = 7). Yoked controls were bred with each generation (N; n = 8). Data referring to offspring is provided in reference to postnatal days, data referring to mothers is provided in reference to GD and postpartum lactational days (LD).

Tissues for miRNA and mRNA expression analyses were collected from representative dams (F0-N, F0-S, F1-NN, F1-SN, F1-SS, F2-SSS, n = 3 per group) on GD21 (placenta) and after weaning of their offspring on LD21 (brain and uterus). From all other dams, uterine tissues were collected on LD21 to count the embryonic implantation sites to account for potential embryonic loss. Left and right uterine horns were pooled for further analyses.

Offspring were sexed at P1 and weighed on P1, P7, P15 and P30. Matched groups of two to three offspring from each sex in each litter were tested in sensorimotor behaviour on P7. Groups of offspring included F1-NN non-stress controls (n = 17), F1-SN stress animals (n = 48), F2-NNN (n = 10), F2-SNN (n = 33), F2-SSN (n = 36), F3-NNNN (n = 10), F3-SNNN (n = 31), F3-SSNN (n = 79) and F3-SSSN (n = 88) animals. Thus, ‘N’ was added to describe the tested offspring generations. Vivarium housing concerns prevented the analysis of pregnancy outcomes for the F3 generation. For the data shown in the figures, male and female animals were combined. All behavioural tests were performed by experimenters blind to the experimental groups.

### Stress procedure

Timed-pregnant rats were stressed daily from GD 12 to GD 18 by restraint and forced swimming. Restraint of the body for 20 minutes occurred between 8:00 and 9:00 AM. Animals were placed in a customized transparent Plexiglas container for a period of 20 minutes each day [20]. The container had perforated ends to allow ventilation. The inner diameter of the container was adjusted to the size of the animals (6 cm inner diameter or larger) to prevent turning and maintain the animals in a standing position without compression of the body. Forced swimming occurred in a round water tank (45 cm diameter, 77 cm high, filled up to 50 cm with 21°C water) for five minutes between 4:00 and 5:00 PM [21].

### Analysis of maternal postpartum behaviour

Postpartum tail chasing behavior was scored as the time spent engaged with the tail and the number of rotations was recorded from 24-hour infrared video recordings [21,22]. Behaviour was videorecorded using an infrared video surveillance system (Panasonic WV-BP330, Panasonic, Minato-ku, Tokyo, Japan). Maternal behaviour was analysed for the first 60 minutes following the delivery of the last pup based on the video recorded data. The amount of time spent engaged in tail chasing and the total number of rotations performed were measured. Initiation of tail chasing behaviour was scored when the dam took interest in her tail followed by chasing or holding the tail with the mouth. Completion of a tail chasing event was scored once the rat disengaged with her tail and initiated a different activity [21]. Tail grooming was not included in this analysis.

### Offspring development

To test proprioceptive, musculoskeletal, and vestibular development on P7, offspring were placed head facing down on a custom-made Plexiglas 40° incline wedge covered with foam tack [23]. Animals were video recorded for one minute and then returned to their mother. The time spent in the downward position until the initiation of a turn was recorded and averaged for three trials.

### Tissue collection

#### Blood

Blood samples (0.6 ml) were collected from the tail vein on GD18 and LD 1 in mothers between 8:00 and 9:00 AM under 4% isoflurane anesthesia [20]. Blood glucose was measured using an Ascensia Breeze Blood Glucose Meter (Bayer, Toronto, ON, Canada) with test strips. The remaining blood was transferred to centrifuge tubes and plasma was obtained by centrifugation at 10,000 rpm for eight minutes. The samples were stored at –20°C. Plasma corticosterone (CORT) levels were determined by enzyme-linked immunosorbent assay (ELISA) using commercial kits (Cayman Chemical, Ann Arbor, MI, USA).

#### Brain, uterus and placenta

Dams received an intraperitoneal overdose of pentobarbital (Euthansol 100 mg/kg; CDMV Inc., Saint-Hyacinthe, QC, Canada). After rapid decapitation, tissues were dissected and flash-frozen for miRNA and transcriptomic analysis. Maternal brain and uterine tissues (n = 3/group) were collected at the time of weaning (three weeks postpartum). Placenta from female offspring was collected from dams (n = 3/group) on GD21.

### mRNA and microRNA expression analysis

#### RNA extraction and microarrays

Total RNA was extracted using TRI Reagent Solution (Applied Biosystems, Foster City, CA, USA). Microarray assay was performed for F0-N, F0-S and F2-SSS frontal cortices using a service provider (LC Sciences, Houston, TX, USA). The assay started from 4 to 8 μg total RNA sample, which was size-fractionated using a YM-100 Microcon centrifugal filter (Millipore, Bedford, MA, USA) and the small RNAs (<300 nt) isolated were 3′-extended with a poly(A) tail using poly(A) polymerase. An oligonucleotide tag was then ligated to the poly(A) tail for later fluorescent dye staining; two different tags were used for the two RNA samples in dual-sample experiments. Hybridization was performed overnight on a μParaflo microfluidic chip using a micro-circulation pump (Atactic Technologies, Houston, TX, USA) [24,25]. On the microfluidic chip, each detection probe consisted of a chemically modified nucleotide coding segment complementary to target miRNA or other RNA (control sequences) and a spacer segment of polyethylene glycol to extend the coding segment away from the substrate. The detection probes were made by *in situ* synthesis using photogenerated reagent (PGR) chemistry. The hybridization melting temperatures were balanced by chemical modifications of the detection probes. Hybridization used 100 μL 6xSSPE buffer (0.90 M NaCl, 60 mM Na2HPO4, 6 mM ethylenediaminetetraacetic acid (EDTA), pH 6.8) containing 25% formamide at 34°C. After RNA hybridization, tag-conjugating cyanine 3 (Cy3) and cyanine 5 (Cy5) dyes were circulated through the microfluidic chip for dye staining. Fluorescence images were collected using a laser scanner (GenePix 4000B, Molecular Device, Sunnyvale, CA, USA) and digitized using Array-Pro image analysis software (Media Cybernetics, Rockville, MD, USA). Data were analysed by first subtracting the background and then normalising the signals using a LOWESS filter (locally-weighted Regression) [26]. For two-color experiments, the ratio of the two sets of detected signals (log2 transformed, balanced) and *P*-values of the *t*-test were calculated. Differentially detected signals were those with *P*-values of less than 0.10.

The putative gene targets for miRNAs were searched by computational analysis (TargetScan, Whitehead Institute for Biomedical Research MIT, Cambridge, MA, USA), which generated a list of predicted gene targets and related biological processes.

#### Quantitative real time PCR

In order to validate miRNAs, we performed quantitative real time PCR (qRT-PCR) analysis of these differentially regulated miRNAs (n = 3 per group for F0, F1 and F2 generations, three replicates per sample): miR-23b, miR-96, miR-141, miR-181a, miR-182, miR-183, miR-200a, miR-200b, miR-200c, miR429 and miR-451. Sno202, U6 and 5 s rRNA were used as references for calculation of the expression ratio. Reverse transcription oligos and amplification primers were designed according to an established protocol [27]. The same samples of total RNA used for microarray analysis were used for qRT-PCR analysis. The generation of cDNAs from the total RNA samples was performed using M-MuLV Reverse Transcriptase, NEB#M0253S (New England Biolab, Ipswich, MA, USA; see Additional file 1: Table S1 for reverse transcription primers). For mRNA quantification, the cDNA were synthesized by using iScript cDNA synthesis kit (Bio-Rad, Mississauga, ON, Canada) following the supplier’s instructions. qRT-PCR reactions were conducted with Bio-Rad CFX96™ Real-Time PCR Systems, using SsoFas™ EvaGreen® Supermix (Bio-Rad) reaction premix added to the cDNAs templates and specific primers [see Additional file 1: Table S1 for primer sequences]. A total volume of 12 μl reaction mix was used, with 2.5 μl of cDNA template, 400 nM forward primer, 400 nM reverse primer and 6 μl of SsoFast™ EvaGreen® Supermix (Bio-Rad).

### Statistical analyses

Phenotypic data, including gestational length, body weight, litter size, glucose, CORT and behavioural data, were analysed using a repeated measures analysis of variance (ANOVA) (group x sex) followed by two-way ANOVA (group x sex) at the different time points. Significant ANOVA results were explored further using either the *post-hoc* Fisher’s least significant difference (LSD) test or the Scheffe test for multiple comparisons. Plasma CORT values were transformed to normality. In addition, correlation analyses used Fisher’s R to Z transformations and Z-tests to calculate correlation coefficients. For all phenotypic data, a *P*-value of less than 0.05 was chosen as the significance level. Analyses were performed using Statview software version 5.0 (SAS Institute, Cary, NC, USA).

For miRNA microarray data *t*-values were calculated with *P*-values below a critical *P*-value (<0.10) selected for cluster analysis, which used a hierarchical method, average linkage and Euclidean distance metric [28]. ANOVA was performed using Bio Rad CFX Manager for validation of miRNA expression by qRT-PCR. All data are presented as mean ± standard error of the mean (SEM).

## Results

### Prenatal and multigenerational stress shortened gestational length across subsequent generations

Compared to the gestational length in non-stress controls (540.37 ± 3.8 hours), stress in the F0-S group did not significantly alter gestational length (537.38 ± 3.3 hours). Both a single generational or multigenerational exposure to prenatal stress modulated gestational length (F(6,34) = 3.48, *P* <0.05). The experience of prenatal stress significantly shortened gestational length in the F1 generation compared to non-stress controls (F(1,15) = 4.48, *P* <0.05; Figure 1B). Of interest, prenatal stress reduced gestational length in F1-SN animals (533.90 ± 2.95 hours) compared to non-stress controls (*P* <0.05). In subsequent generations, gestational length was further shortened by a stressful pregnancy in the F1-SS group (529.30 ± 0.87 hours, *P* <0.01) and among all groups that were exposed to prenatal and/or gestational stress (F(2,23) = 6.75, *P* <0.05; Figure 1B) in the F2 generation. In particular, non-stressed F2 dams whose grandmothers were stressed during gestation (F2-SNN; 530.10 ± 1.1) had a significantly shorter gestational length compared to that of non-stress controls (*P* <0.001). F2 dams whose grandmothers and mothers were stressed (F2-SSN; 528.2 ± 1.42 hours) or were stressed in each generation (F2-SSS; 527.78 ± 0.9 hours) also had shorter gestational lengths than those of non-stress controls (*P* <0.001).

### Prenatal stress elevated blood glucose levels

The reductions in gestational lengths were accompanied by altered blood glucose concentrations in late pregnancy and postpartum. There was an overall effect of stress on glucose levels (F(3,33) = 3.71, *P* <0.05). In the F2 generation, SSN animals had higher basal blood glucose levels than non-stress F2-NNN (*P* <0.05) and F2-SSS (*P* <0.05) rats on GD 18 (Figure 1C). Furthermore, non-stressed dams whose grandmothers and mothers were stressed (F2-SSN) displayed higher blood glucose levels on GD18 (5.06 ± 0.25) compared to F2-NNN controls (4.0 ± 0.39, *P* <0.05; Figure 1C). Multigenerational stress in F2-SSS animals caused elevated gestational blood glucose levels on GD18 when compared to F2-NNN rats (5.19 ± 0.36, *P* <0.05) and after birth on LD 1 compared to F2-SNN and F2-SSN rats (7.0 ± 0.22, all *P*s <0.05). Plasma CORT levels revealed that F2-SSS dams on GD18 had higher CORT levels than any F0 parental group (F2-SSS versus F0-S *P* <0.001, F2-SSS versus controls *P* <0.01).

### Stress reduced gestational weight gain

**Figure 2:**
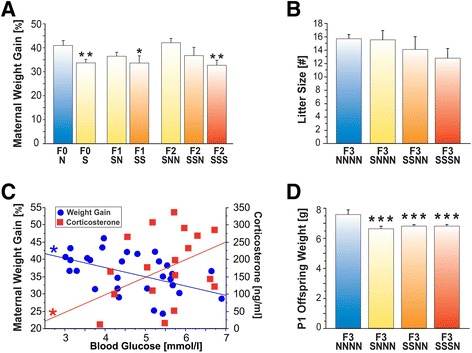
**Gestational stress and prenatal stress reduce gestational weight gain and birth weight in the absence of litter size reduction. (A)** Gestational stress reduced maternal weight gain during pregnancy. Maternal weight gain was mainly affected by gestational stress (F0-S) or cumulative effects of multigenerational stress (F1-SS, F2-SSS). **(B)** Ancestral stress did not affect litter size. **(C)** Higher blood glucose values were associated with reduced maternal weight gain during pregnancy and elevated corticosterone levels on lactational day 1. **(D)** Transgenerational and multigenerational prenatal stress resulted in low birth weight among F3 offspring. Asterisks indicate significances: *P <0.05; **P <0.01; ***P <0.001, compared to the respective generational non-stress controls.

In the absence of changes in litter size, stress during gestation reduced gestational weight gain in pregnant dams. Weight gain was analysed as a percentage of change compared to pre-gestational body weight. On GD11, F2-SSN and F2-SSS dams weighed on average 20 g less than F2-NNN dams (*P*s <0.05). On GD21, the F0-S dams were lighter than F0-N dams (*P* <0.01) and F1-SS lighter than F1-SN and F1-NN (*P* <0.05), In the F2 generation, F2-SSS dams were lighter than F2-NNN (*P* <0.001) and F2-SNN dams (*P* <0.05; Figure 2A). There was no difference in litter size between groups, however (Figure 2B). There were no differences in uterine implantation sites between the groups in each generation; however, F2-SSN dams showed significantly more sites than F0-S and F1-SS dams (all *P*s <0.05) in the absence of litter size differences. Notably, dams that showed higher blood glucose levels on LD1 also showed lower gestational weight gain (r = 0.36, *P* <0.05; Figure 2C) and elevated CORT levels on GD21 (r = 0.44, *P* <0.05; Figure 2C).

### Prenatal and transgenerational stress impeded offspring growth trajectories

In the developing offspring, prenatal stress delayed growth trajectories. There was an effect of group (F(1,8) = 6.176, *P* <0.001) and sex (F(1,8) = 64.756, *P* <0.001), but no interaction between the two factors. The effects of prenatal stress on offspring weight in the F1 generation became evident by P7 (F(1,64) = 57.97, *P* <0.0001), with F1-SS offspring (n = 48) being 3 g lighter than F1-NN controls (n = 17, *P* <0.001). This difference in weight remained significant into adulthood. Within each group and at all ages, female offspring were always lighter than male offspring (*P* <0.01).

Notably, the effects of prenatal stress on body weight were not noticeable at P1 until the F3 generation (see Figure 2D). All stress-treated F3 offspring groups were different from controls (F(3,241) = 5.12, *P* <0.001). Hence, the F3-SNNN (n = 31), F3-SSNN (n = 79) and F3-SSSN (n = 88) groups showed significant weight reduction of about 0.5 g compared to F3-NNNN animals (n = 44, all *P*s <0.001). In F3-SNNN and F3-SSNN groups these effects remained significant throughout P7 (*P* <0.001), while F3-SSSN animals were not different from their F3-NNNN peers. At P15, this pattern was quite similar, revealing that the growth rate of F3-SSNN (*P* <0.001) and F3-SNNN (*P* <0.05) groups stayed behind that of F3-SSSN or F3-NNNN animals. Further, transgenerationally stressed F3-SNNN animals were lighter than their F3-SSNN counterparts (*P* <0.001) while multigenerationally stressed F3-SSSN animals were not different from F3-NNNN rats. At P30, F3-SSNN offspring were still lighter than F3-SSSN or F3-NNNN groups (*P* <0.001). Within all groups and at all ages, females were always lighter than males (*P* <0.01). Correlation analysis revealed that there was no influence of litter size on developmental trajectories.

### Prenatal stress modified maternal behaviour across generations

**Figure 3:**
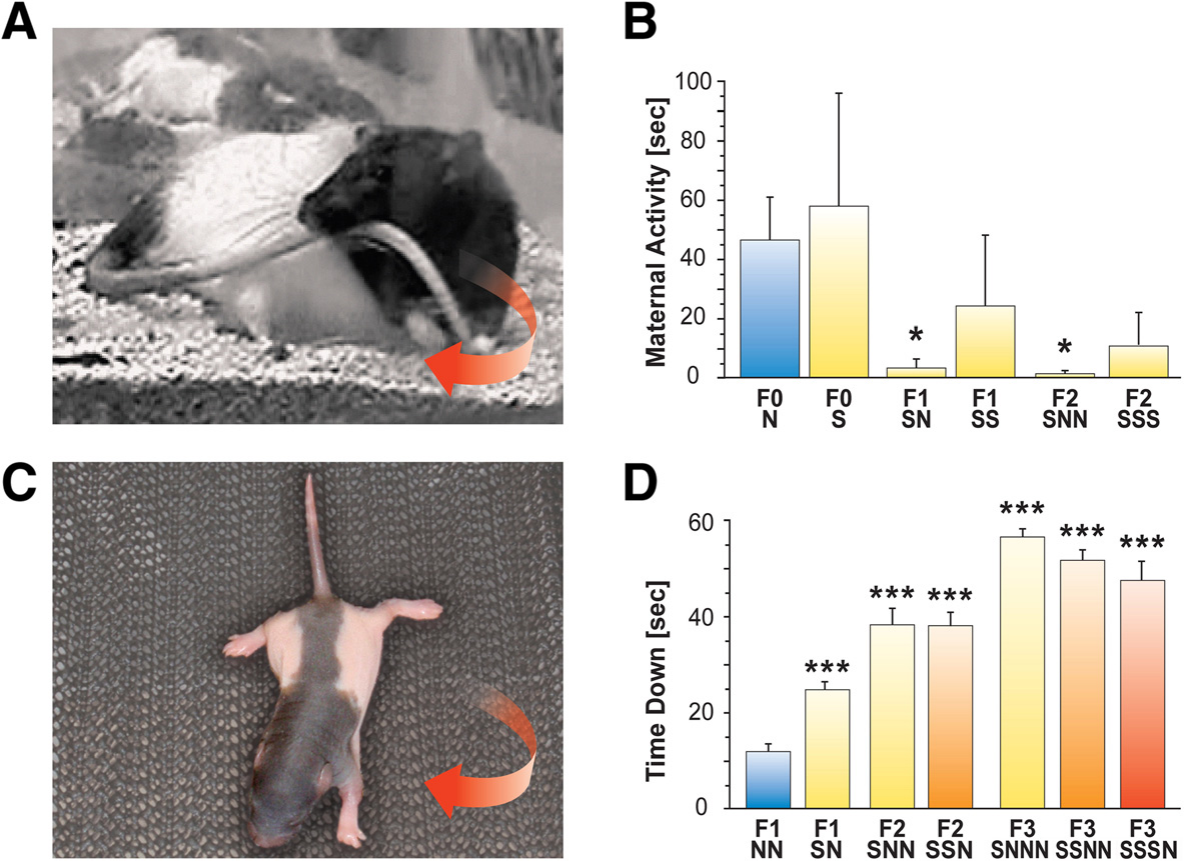
**Ancestral stress alters maternal behaviour and offspring sensorimotor development. (A)** Illustration of a dam carrying her tail during rotations in recordings of postpartum maternal activity. **(B)** Time spent in tail chasing behaviour during the first hour after completed delivery of her offspring. Note that a history of prenatal stress reduced maternal tail chasing activity. **(C)** Photograph of offspring performing the inclined plane test on postnatal day 7. Pups were placed head facing down on an inclined plane. **(D)** Latency to respond with a rotating movement in seven-day old pups. Note that a history of prenatal stress delayed the turning response across generations. Asterisks indicate significances: *P <0.05; ***P <0.001, compared to the respective generational non-stress controls.

At one hour following delivery, the profile of motor activities in dams was used as an indicator of gestational and prenatal stress. There were no differences in the parental generation between non-stressed controls and stressed dams. Overall, the experience of prenatal stress reduced tail chasing across groups (*P* <0.01), which was further reduced in the F2 generation (*P* <0.001). In the F1 generation, tail chasing and rotational behaviours (Figure 3A) of F1-SN dams were reduced compared to F0-N controls (*P* <0.05, Figure 3B). In the F2 generation, SNN dams spent significantly less time in tail chasing compared to controls (*P* <0.01).

### Prenatal and transgenerational stress delayed offspring sensorimotor development

Proprioceptive, musculoskeletal, and vestibular development on P7 revealed an overall difference between groups (F(8,341) = 20.39, *P* <0.001). There was no effect of sex and no interaction between group and sex. Prenatally stressed F1-SN pups (n = 48), compared to non-stressed F1-NN pups (n = 17), showed a significantly delayed turning response (Figure 3C) when placed on an inclined plane (*P* <0.001; Figure 3D). The F2-SNN (n = 33) and F2-SSN pups (n = 36) showed further prolonged latencies compared to their F2-NNN counterparts (n = 10; *P* <0.001). The F3 generation showed a longer latency after grandmaternal stress in F3-SNNN animals (n = 31; *P* <0.001) and in F3-SSNN animals whose grandmothers and mothers were stressed (n = 79; *P* <0.001) or in F3-SSSN animals where all three generations experienced stress (n = 88; *P* <0.001; Figure 3D). These observations indicate that sensorimotor impairments resulted in slower response times.

### MicroRNA (miRNA) profiles were altered in the F2 progenies of stressed animals

**Figure 4:**
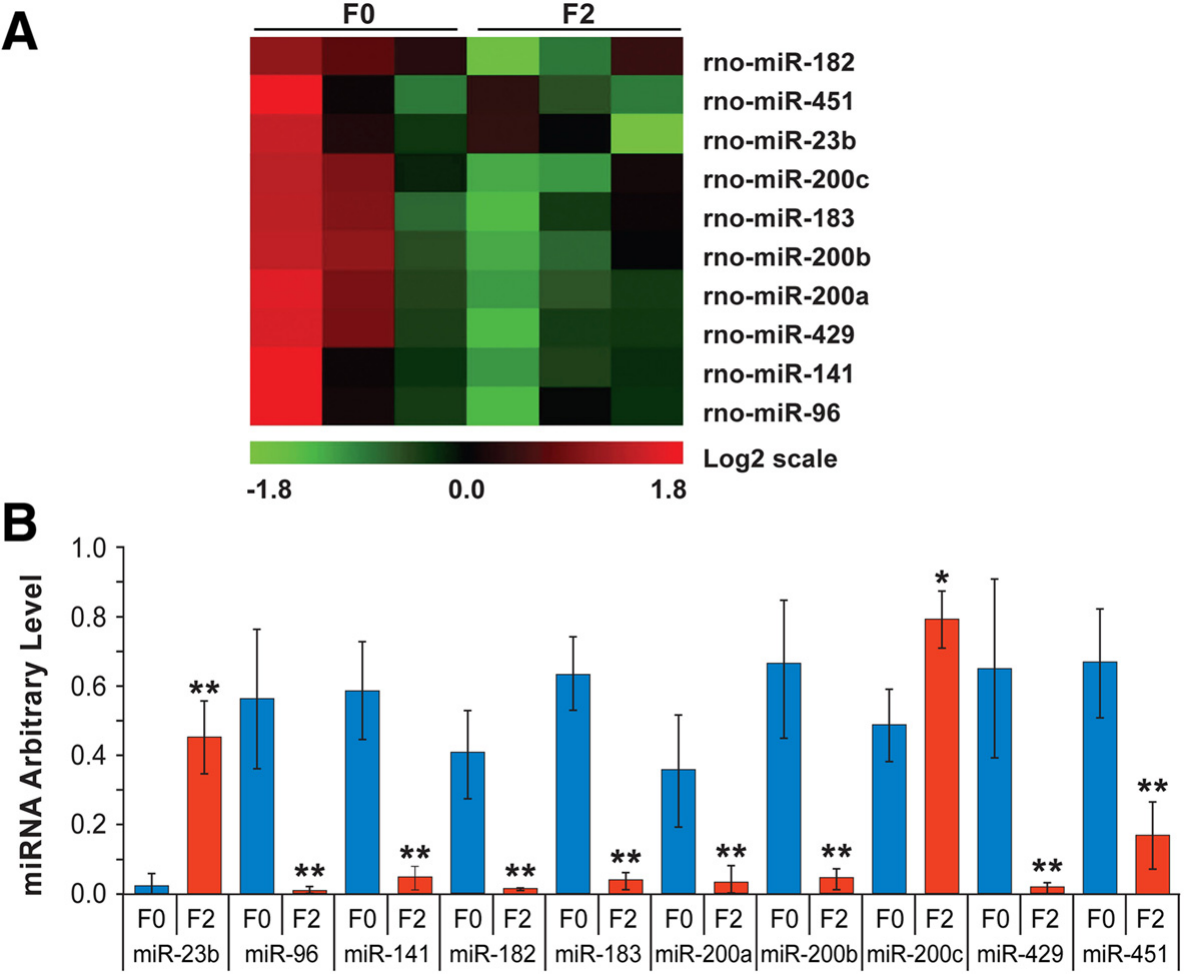
**Ancestral stress alters brain miRNA expression. (A)** Heat map of miRNA expression modulated by multigenerational stress in brains of F2-SSS dams. **(B)** Confirmation of miRNA level changes in brains of F0-S and F2-SSS compared to non-stress F0-N rats by qRT-PCR. Ancestral programming by stress particularly involved the miR-200 family. Sno202, U6 and 5 s rRNA were used as references. Asterisks indicate significances: *P <0.05; **P <0.01, compared to F0-S levels.

In consideration of the possible role of miRNA-mediated stress adaptation, we profiled miRNA from the frontal cortices of F0-N, F0-S and F2-SSS animals using a microarray-based approach. Compared to F0-N controls, rno-miR-138-1-3p* was significantly induced in the frontal cortex of F0-S dams whereas rno-miR-323-5p was significantly suppressed (*P* <0.01, n = 3). In addition, compared to F0-N rats, stress in F0-S dams induced one miRNA (rno-miR-466b-1-3p) and suppressed the expression of three miRNAs (rno-miR-145-3p, rno-miR-24-1-5p and rno-miR-375) (all *P*s <0.10). Interestingly, ten miRNAs showed significant changes (*P* <0.10) between non-stressed F0-N and F2-SSS brains. However, since the signal level of these miRNAs was relatively low, we chose an additional set of miRNAs, which demonstrated high signal level and significantly altered expression levels based on *t*-test comparisons for qRT-PCR validation (Figure 4A). The qRT-PCR confirmed changes of the selected miRNAs (Figure 4B), decreased expression of miR-96, miR-141, miR-182, miR-183, miR-200a, miR-200b, miR-429 and miR-451 in F2-SSS compared to F0-S animals, whereas miR-23b and miR-200c showed increased expression levels. Thus, multigenerational stress in the F2-SSS cortex modulated miRNA profiles.

### Target genes of altered miRNAs in brain include transcription regulators and mediators of neuropsychiatric disorders and endocrine pathways

Based on the validated list of altered miRNAs in the brain (frontal cortex), we compiled putative target genes using the mirSVR predicted target site scoring method [29]. To explore the biological processes involving the putative gene targets, functional classification tools from DAVID [30] were used. Results are summarized in Supplementary Material [see Additional file 1: Table S1]. Between 17.1% to 23.7% of putative target genes were transcription regulators and an additional 2.3% to 5.5% were related to chromatin organization. Furthermore, a significant number of target genes assume roles in the genesis, reception or processing of endocrine functions including hormones, insulin, vitamins, carbohydrates, nutrients and drugs, or in embryonic development.

Possible involvement of miRNAs in disease pathways was suggested by the classification of putative gene target lists using PANTHER [31]. Supplementary material [see Additional file 2: Table S2] summarizes the potential of these miRNA target genes that affect known disease pathways of metabolic, physiological, inflammatory, immunological, oncological, developmental and neuropsychiatric disorders.

### Multigenerational programming by stress modulated uterine miRNA and gene expression involved in preterm birth

**Figure 5:**
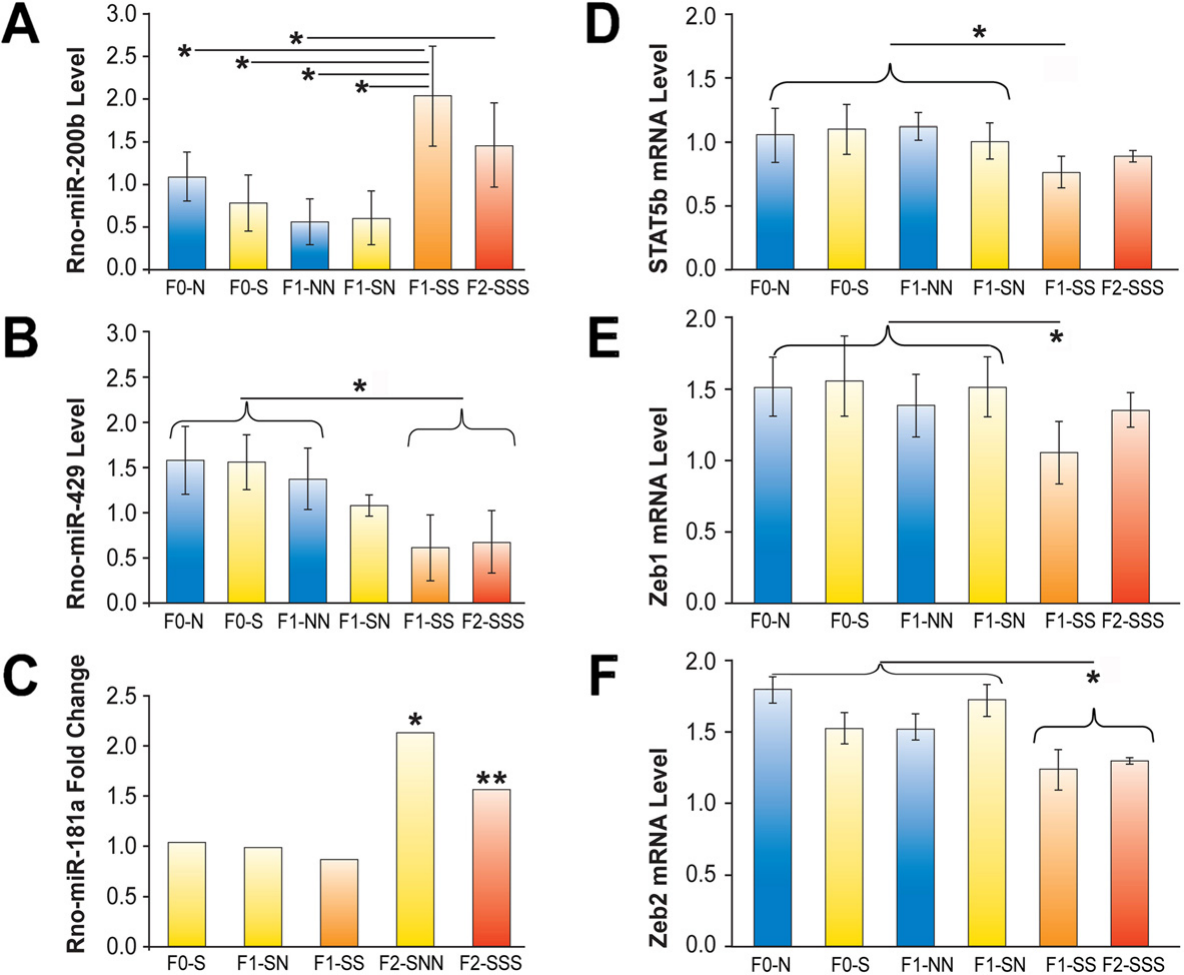
**Ancestral stress alters expression of miRNA and their target genes in the uterus and placenta. (A)** Arbitrary maternal uterine miR-200b expression levels across F0, F1 and F2 generations (n = 3). Multigenerational stress in the F1-SS and the F2-SSS generations elevated miR-200b expression levels. Sno202, U6 and 5 s rRNA were used as references. **(B)** Arbitrary maternal uterine miR-429 expression levels across F0, F1 and F2 generations. Multigenerational stress in the F1 and F2 generations downregulated miR-429 expression. **(C)** Fold change of placental miR-181a expression in female offspring. Ancestral stress elevated miR-181a expression in female offspring in the F2 generation, but not in F1 animals. **(D-F)** Arbitrary uterine *Stat5b*, *Zeb1* and *Zeb2* mRNA levels across F0, F1 and F2 generations. Stress reduced *Stat5b*, *Zeb1* and *Zeb2* gene expression in the F1-SS generation. Reduced *Zeb2* gene expression also occurred in the F2-SSS generation **(F)**. GAPDH was used as a reference. Asterisks indicate significances: *P <0.05; **P <0.01, compared to the respective non-stress controls. miRNA, microRNA.

Cumulative multigenerational stress upregulated miR-200b and downregulated miR-429 expression levels in the uterus of F1-SS and F2-SSS generations (Figures 5A, B). Both miR-200b and miR-429 are known to modulate gestational length through interaction with their target genes *Stat5b*, *Zeb1* and *Zeb2* [18]. When upregulated, miR-200b may act to suppress *Stat5b*, *Zeb1* and *Zeb2* mRNA levels in the F1-SS and F2-SSS generations (Figures 5D-F), while reduced *Zeb2* expression in particular was transmitted to the F2-SSS generation (Figure 5F). The findings suggest that miR-429 may not have a suppressive role on *Stat5b*, *Zeb1* and *Zeb2* in postpartum dams.

### Stress across generations modified placental microRNA predictors of preterm birth

MiR-181a, which is altered in placentas of human preterm birth [32], remained unaltered in stressed F0 and F1 generations (Figure 5C). In the F2-SNN and F2-SSS groups, however, miR-181a was significantly upregulated compared to F2-NNN animals (n = 3, *P* <0.001 and *P* <0.01, respectively; Figure 5C), indicating programming by the cumulative effects of stress.

## Discussion

In about half of human cases, the causes of PTB remain unknown. Here, we provide evidence that gestational stress across generations of timed-pregnant rats has downstream effects on endocrine, metabolic and behavioural manifestations of PTB, and leads to shortened gestational length. Developmental trajectories across all generations of offspring were affected as early as P7. In terms of molecular mechanisms, stress in the parental F0 generation had minor effects on regulatory miRNA pathways in brain, uterus and placenta. By contrast, a history of stress in the F2 generation was associated with drastic changes in somatic tissue miRNA profiles and altered expression of genes that have been linked to PTB in humans. Notably, genuine transgenerational programming of developmental trajectories was observed in the F3 generation, in which gestational stress was imposed on the great-grandmaternal generation and was inherited to affect the developing embryo. These findings suggest that the mechanisms involved in the timing of parturition and associated behavioural and physiological signatures can be programmed through the maternal lineage.

A main finding of the present study is that gestational length is influenced by prenatal stress rather than by gestational stress. The impact of prenatal stress on phenotype in the present study was illustrated by delayed developmental milestones in the F1 generation, with an even stronger impact in subsequent F2 and F3 generations. Prenatal stress has been shown to program fetal brain development, HPA axis function and mental health [33–35]. Thus, early experiences can prime physiological and immunological processes that may lead to variations in gestational length [36,37] and susceptibility to altered glucose metabolism, such as type 2 diabetes [38] in adulthood. The timing and severity of the stressor is critical in that stress in early pregnancy may have greater effects on health outcomes than stress experienced in the last trimester [39]. The present study induced stress from gestational days 12 to 18, representing a period thought to cover a large extent of the human second trimester [40,41] and in rats has been shown to be particularly susceptible to environmental influences, inflammatory processes and stress [42]. While the present study controlled for adverse effects of stress, other factors, such as anesthetic administration, may still have affected the present outcomes [43]. Altogether, it is possible that gestational and inter-generational programming of HPA axis responses may sensitize the response to environmental adversity thus resulting in gradually shortened gestation across generations and further reduction in the multigenerationally stressed cohorts (SSN and SSS).

Beyond fetal endocrine programming by an altered gestational endocrine milieu, maternal distress during pregnancy may also critically affect offspring brain development and physiology through variation in maternal behaviours [21,22,44]. The present data show that prenatal stress alters patterns of early postpartum maternal behaviours, which may be predictive of altered maternal care and stress coping at later times. The first hour after completed parturition may represent a critical transition phase in which the characteristic patterns of late antepartum behaviours, including tail chasing activity and nest building, convert into maternal care of the offspring [21]. It has been demonstrated that the early postnatal environment, such as variations in maternal care, determines developmental and epigenetic outcomes [45,46]. Patterns of altered maternal behaviour may transmit to subsequent generations [21,47]. The contribution of endocrine and behavioural influences to generational programming is complex and likely reciprocally regulated by the epigenome.

In line with previous findings of stress-induced alteration in brain miRNA profiles [48] and according to the present observations of altered maternal behaviour, multigenerational stress in the F2-SSS group had prominent effects on miRNA expression patterns in the frontal cortex. Interestingly, F2-SSS dams showed upregulated miR-23b, which regulates oligodendrocyte development and myelination [49]. miR-200 family members, including the downregulated miR-200a, are predicted to target genes that regulate synaptic function, neurodevelopment and neuronal survival [50]. Stress also downregulated miRNAs that possess potential roles in the pathogenesis of psychiatric diseases, such as miR-96 [51], miR-182 and miR-183 [52]. Furthermore, stress-induced downregulation concerned miR-429, which potentially influences development by altering cell proliferation and apoptosis [53]. It is important to note that many neurodegenerative and psychiatric disorders share a pathology involving miRNA regulation [22,54,55] and that these miRNAs may in turn regulate central stress responses [54]. Though not determined in this study, it is likely that these miRNA changes are not limited to the prefrontal cortex and uterus, thus indicating a potential intersection linking psychological stress to altered gestational length.

Across all generations, mechanisms of prenatal stress to modulate gestational length may include modulation of the complex pro-inflammatory state leading to PTB [37]. Furthermore, stress may affect levels of hormones and neuropeptides, including prolactin, progesterone and oxytocin, which are involved in maintenance of pregnancy and timing of delivery [36]. Increased fetal HPA axis activity may induce prostaglandin production by fetal membranes and decidua leading to uterine activation [56]. In addition, stress may stimulate cytokines, which regulate the activity of placental 11-beta-hydroxysteroid dehydrogenase [57] to elevate PTB risk. These endocrine regulations have led to the notion that PTB risk may have roots in childhood [6]. The present data confirm this notion and provide possible mechanistic links to epigenetic regulation of gene expression related to PTB risk.

Including the downregulated miR-200b, the miR-200 family may exert peripheral effects to control uterine quiescence and contractility during pregnancy and labour [18]. Interestingly, miR-200b/200c/429 are induced at term labour in mice and humans and miR-200b/200c/429 are upregulated in mouse models of preterm labour [18]. This group of miRNAs may largely interact with the endocrine cascade involved in pregnancy maintenance and termination, including progesterone and oxytocin [18]. Moreover, miR-451 is expressed in the uterus [58,59] and regulated by estrogen and progesterone [58].

Target genes of the miR-200 family include three particular genes, *Stat5b*, *Zeb1* and *Zeb2*, all involved in pregnancy maintenance [18]. In the uterus, all three were downregulated by multigenerational stress in the F1 generation. Effects on *Zeb2* expression were transmitted to the F2 generation. These findings concur with the reduction in gestational length. Accordingly, a decrease in *Stat5b* expression was linked to reduced progesterone activity and the initiation of labor, in particular in preterm birth [19]. Furthermore, ZEB1 serves as transcription factor to inhibit the miR-200 family, thus enhancing *Stat5b* expression [19]. As the myometrium transitions to term or preterm labor, reduced progesterone activity decreases ZEB1 and ZEB2 levels via a feed-forward mechanism [18,19], thus regulating the timing of parturition. The upregulation of uterine miR-200b may be causative for the suppression of *Stat5b* and ZEB1 and ZEB2; however, they may also reflect low postpartum progesterone levels due to timing of tissue sampling in the present study. Although the direction of these and the placental miR-181a changes are opposed to the downregulation found in human preterm birth [32], their differential expression across generations coincides with shortened gestational length and indicates a causal or, at least, predictive signature of preterm birth.

A role for genuine epigenetic inheritance of stress response is suggested by the present findings concerning the F3 generation. While context-dependent programming may have mainly determined the F1 and F2 phenotype, programming of the germ-line became evident by altered development in the F3 generation [16]. Indeed, the most dramatic impact of prenatal stress on developmental trajectories was found in the F3 generation. Only in the F3 generation did the offspring display low body weight already on P1, which was associated with reduced growth trajectories and a drastic sensorimotor behaviour deficit. Since these phenotypic changes persisted to the F3-SNNN generation in the absence of direct somatic exposure, they are arguably mediated by genuine transgenerational programming of the female germline [16,17,60]. This suggests transgenerational epigenetic inheritance whereby the epigenetic modifications may have been passed on via the gametes that have escaped reprogramming [16,61,62]. Thus, the study of transgenerational programming of epigenetic signatures may provide a unique opportunity to identify predictive biomarkers and future therapeutic targets to promote maternal and child health.

## Conclusions

The present findings show that prenatal stress is associated with an increased risk of shortened gestational length, poor pregnancy outcomes and delayed offspring development. Results from this study suggest that: 1) the mechanisms involved in the timing of parturition are vulnerable during early development; 2) there is a compounding effect of gestational stress on physiological and behavioural outcomes that propagate across subsequent generations; and 3) that these changes are accompanied by altered miRNA regulation in somatic cells. The identification of stress-induced epigenetic signatures in clinically accessible tissues, such as the placenta, offers an exciting potential for the prediction and prevention of PTB and poor pregnancy outcomes. The present findings concur with descriptions of inter-generational stress impacts by human migration, natural disasters and poverty, which may program maternal health preconceptionally via the maternal lineage. Although spontaneous PTB in humans is likely a multifactorial condition, the present data offer a potentially clinically relevant platform to study predictive factors and interventions for PTB and adverse developmental outcomes.

## Additional files

Additional file 1: Table S1. Percentage of genes participating in biological pathways that affect gestation. Additional file 2: Table S2. Number of target genes involved in pathways screened by PANTHER (red font denotes pathways in neuropathology).

## Abbreviations

ANOVA: analysis of variance
CORT: corticosterone
GD: gestational day
LD: lactational day
HPA: hypothalamic-pituitary-adrenal
miRNA: microRNA
N: non-stress
P: postnatal day
PTB: preterm birth
qRT-PCR: quantitative real time-polymerase chain reaction
S: stress
SN: non-stressed
