# Supplementary material for: Ancestral exposure to stress epigenetically programs preterm birth risk and adverse maternal and newborn outcomes
Source: BMC Med. 2014 Aug 7;12:121. doi: 10.1186/s12916-014-0121-6 (PMC4244860; doi:10.1186/s12916-014-0121-6)
Supplement: Additional file 1: Table S1. — Percentage of genes participating in biological pathways that affect gestation. [file 12916_2014_121_MOESM1_ESM.docx]

| ***Table S1***. Percentage of genes participating in biological pathways that affect gestation. | | | | | | | | |
| --- | --- | --- | --- | --- | --- | --- | --- | --- |
| **Biological Process** | **mir-141/200a** | **mir-200bc/429** | **mir-183** | **mir-9** | **mir-23b** | **mir-103a** | **mir-329** | **mir-96** |
| Total target gene number | 517 | 758 | 283 | 943 | 722 | 403 | 218 | 784 |
|  |  |  |  |  |  |  |  |  |
| regulation of transcription | **22.8 | 23.7 | 20.5 | 20.6 | 23.0 | 21.1 | 20.6 | 17.1 |
| chromatin organization | 3.7 | 3.7 | 2.5 | 3.6 | 4.2 | 2.5 | 5.5 | 2.3 |
| response to organic substance* | 6.0 | 7.0 | 5.7 | 4.6 | 5.1 | 3.2 | 3.2 | 5.0 |
|  |  |  |  |  |  |  |  |  |
| chordate embryonic development | 3.7 | 4.0 | 4.6 | 3.4 | 2.9 | 4.0 | 2.8 | 4.5 |
| embryonic appendage morphogenesis | 1.4 | 0.5 | 2.1 | 1.0 | 0.8 | 0.7 | 1.8 | 1.3 |
| embryonic epithelial tube formation | 1.2 | 0.7 | n/a | 0.7 | 0.7 | n/a | n/a | 1.0 |
| embryonic limb morphogenesis | 1.4 | 0.5 | 2.1 | 1.0 | 0.8 | 0.7 | 1.8 | 1.3 |
| embryonic morphogenesis | 4.3 | 3.7 | 4.2 | 2.9 | 4.6 | 3.2 | 5.5 | 4.3 |
| embryonic organ development | 1.4 | 2.9 | 2.5 | 1.4 | 2.6 | 1.7 | 2.3 | 0.0 |
| embryonic organ morphogenesis | n/a | 1.7 | 1.4 | 0.8 | 2.2 | 1.5 | 2.3 | 1.4 |
| embryonic skeletal system development | 1.4 | 1.1 | n/a | 0.8 | 1.5 | 1.0 | n/a | n/a |
| embryonic skeletal system morphogenesis | 1.0 | 0.8 | n/a | 0.5 | 1.4 | 1.0 | n/a | n/a |
| in utero embryonic development | 1.4 | 2.4 | 2.5 | 1.9 | n/a | 2.5 | 1.4 | 2.6 |
| morphogenesis of embryonic epithelium | 1.4 | 0.9 | n/a | 0.8 | 0.7 | n/a | n/a | 1.1 |
|  |  |  |  |  |  |  |  |  |
| blood vessel development | 1.9 | 1.8 | 2.5 | 2.4 | 2.4 | 2.5 | 1.4 | 2.7 |
| blood vessel morphogenesis | 1.7 | 1.3 | 1.8 | 2.0 | 2.5 | 2.2 | 1.4 | 1.9 |
| Note: *: organic substances include hormones, insulin, vitamins, carbohydrates, nutrients and drugs | | | | | | | | |
| **: numbers reflect percentage of total number of target genes. | | | | | | | | |
