# Supplementary material for: Ancestral exposure to stress epigenetically programs preterm birth risk and adverse maternal and newborn outcomes
Source: BMC Med. 2014 Aug 7;12:121. doi: 10.1186/s12916-014-0121-6 (PMC4244860; doi:10.1186/s12916-014-0121-6)
Supplement: Additional file 2: Table S2. — Number of target genes involved in pathways screened by PANTHER (red font denotes pathways in neuropathology). [file 12916_2014_121_MOESM2_ESM.docx]

| ***Table S2***. Number of target genes involved in pathways screened by PANTHER. | | | | | | |  |  |  |  |
| --- | --- | --- | --- | --- | --- | --- | --- | --- | --- | --- |
| Path ID* | Pathway name | **mir-141 /200a** | **mir-200bc /429** | **mir-96** | **mir-183** | **mir-23b** | **mir-9** | **mir-103a** | **mir-329** | **mir-182** |
| P00057 | Wnt signaling pathway | 18 | 19 | 20 | 20 | 36 | 26 | 13 | 23 | 16 |
| P00018 | EGF receptor signaling pathway | 13 | 17 | 18 | 8 | 15 | 17 | 7 | 3 | 17 |
| P00036 | Interleukin signaling pathway | 13 | 19 | 18 | 3 | 13 | 18 | 3 | 2 | 15 |
| P00031 | Inflammation mediated by chemokine and cytokine signaling pathway | 12 | 19 | 17 | 9 | 15 | 14 | 10 | 4 | 18 |
| P00048 | PI3 kinase pathway | 12 | 18 | 18 | 6 | 13 | 11 | 5 | 2 | 16 |
| P00052 | TGF-beta signaling pathway | 12 | 18 | 18 | 4 | 15 | 17 | 4 | 3 | 15 |
| P00033 | Insulin/IGF pathway-protein kinase B signaling cascade | 11 | 12 | 13 | 6 | 12 | 12 | 3 | 2 | 10 |
| P00049 | Parkinson’s disease | 11 | 11 | 10 | 1 | 9 | 4 | 5 | 5 | 8 |
| P00012 | Cadherin signaling pathway | 10 | 8 | 12 | 11 | 20 | 8 | 5 | 18 | 9 |
| P00021 | FGF signaling pathway | 10 | 17 | 16 | 8 | 10 | 13 | 7 | 2 | 15 |
| P00034 | Integrin signalling pathway | 10 | 19 | 22 | 4 | 14 | 25 | 9 | 4 | 13 |
| P00059 | p53 pathway | 10 | 13 | 5 | 4 | 11 | 7 | 3 | 3 | 4 |
| P00005 | Angiogenesis | 6 | 19 | 18 | 7 | 8 | 17 | 7 | 5 | 16 |
| P00029 | Huntington’s disease | 6 | 17 | 8 | 1 | 4 | 14 | 7 | 5 | 9 |
| P00039 | Metabotropic glutamate receptor group III pathway | 6 | 8 | 9 | 3 | 3 | 7 | 5 | 4 | 9 |
| P00047 | PDGF signaling pathway | 6 | 24 | 14 | 5 | 12 | 21 | 4 | 4 | 16 |
| P00004 | Alzheimer’s disease-presenilin pathway | 5 | 8 | 4 | 5 | 6 | 14 | 6 | 5 | 4 |
| P00025 | Hedgehog signaling pathway | 5 | 9 | 3 | 2 | 4 | 5 | 4 | 2 | 3 |
| P00027 | Heterotrimeric G-protein signaling pathway-Gq alpha and Go alpha mediated pathway | 5 | 6 | 11 | 7 | 7 | 6 | 8 | 3 | 7 |
| P00032 | Insulin/IGF pathway-mitogen activated protein kinase kinase/MAP kinase cascade | 5 | 4 | 9 | 2 | 1 | 6 | 0 | 1 | 8 |
| P00037 | Ionotropic glutamate receptor pathway | 5 | 2 | 4 | 1 | 2 | 8 | 4 | 5 | 6 |
| P00006 | Apoptosis signaling pathway | 4 | 13 | 9 | 2 | 11 | 7 | 3 | 1 | 6 |
| P00010 | B cell activation | 4 | 4 | 7 | 0 | 3 | 6 | 2 | 1 | 8 |
| P00015 | Circadian clock system | 4 | 3 | 2 | 1 | 2 | 2 | 2 | 2 | 2 |
| P00019 | Endothelin signaling pathway | 4 | 9 | 10 | 4 | 5 | 6 | 6 | 2 | 10 |
| P00046 | Oxidative stress response | 4 | 6 | 7 | 4 | 3 | 4 | 3 | 2 | 6 |
| P00003 | Alzheimer’s disease-amyloid secretase pathway | 3 | 5 | 4 | 3 | 2 | 9 | 5 | 1 | 4 |
| P00007 | Axon guidance mediated by semaphorins | 3 | 6 | 6 | 3 | 0 | 4 | 5 | 4 | 4 |
| P00026 | Heterotrimeric G-protein signaling pathway-Gi alpha and Gs alpha mediated pathway | 3 | 12 | 8 | 3 | 10 | 7 | 7 | 4 | 9 |
| P00044 | Nicotinic acetylcholine receptor signaling pathway | 1 | 6 | 14 | 1 | 3 | 12 | 3 | 2 | 8 |
| P00016 | Cytoskeletal regulation by Rho GTPase | 3 | 12 | 8 | 5 | 6 | 8 | 2 | 1 | 7 |
| P00008 | Axon guidance mediated by Slit/Robo | 3 | 3 | 2 | 2 | 3 | 6 | 1 | 0 | 2 |
| P00035 | Interferon-gamma signaling pathway | 3 | 5 | 2 | 2 | 3 | 2 | 2 | 2 | 3 |
| P00041 | Metabotropic glutamate receptor group I pathway | 3 | 2 | 5 | 1 | 1 | 0 | 1 | 1 | 3 |
| P00042 | Muscarinic acetylcholine receptor 1 and 3 signaling pathway | 3 | 4 | 8 | 5 | 2 | 5 | 3 | 1 | 5 |
| P00040 | Metabotropic glutamate receptor group II pathway | 2 | 6 | 5 | 3 | 0 | 5 | 5 | 2 | 5 |
| P00055 | Transcription regulation by bZIP transcription factor | 1 | 7 | 4 | 0 | 1 | 3 | 1 | 0 | 4 |
| P00043 | Muscarinic acetylcholine receptor 2 and 4 signaling pathway | 2 | 7 | 5 | 3 |  | 4 | 4 | 1 | 4 |
| P00053 | T cell activation | 2 | 7 | 8 | 1 | 4 | 6 | 1 | 1 | 8 |
| P00030 | Hypoxia response via HIF activation | 1 | 6 | 3 | 1 | 6 | 3 | 2 | 2 | 2 |
| P00056 | VEGF signaling pathway | 2 | 7 | 6 | 3 | 4 | 7 | 2 | 1 | 6 |
| P00001 | Adrenaline and noradrenaline biosynthesis | 0 | 3 | 3 | 0 | 1 | 6 | 1 | 1 | 3 |
| P00060 | Ubiquitin proteasome pathway | 0 | 6 | 4 | 0 | 3 | 6 | 1 | 0 | 5 |
| P00009 | Axon guidance mediated by netrin | 1 | 2 | 5 | 1 | 2 | 6 | 1 | 1 | 5 |
| P00054 | Toll receptor signaling pathway | 2 | 3 | 3 | 0 | 2 | 2 | 0 | 0 | 3 |
| P00002 | Alpha adrenergic receptor signaling pathway | 1 | 2 | 2 | 2 | 3 | 4 | 3 | 2 | 3 |
| P00045 | Notch signaling pathway | 1 | 3 | 2 | 2 | 1 | 5 | 3 | 0 | 4 |
| P00020 | FAS signaling pathway | 1 | 4 | 1 | 0 | 2 | 0 | 0 | 0 | 1 |
| P04393 | Ras Pathway | 5 | 13 | 10 | 4 | 0 | 15 | 0 | 1 | 9 |
| P04374 | 5HT2 type receptor mediated signaling pathway | 1 | 6 | 12 | 6 | 3 | 8 | 5 | 1 | 10 |
| P04391 | Oxytocin receptor mediated signaling pathway | 1 | 6 | 11 | 6 | 2 | 5 | 4 | 1 | 9 |
| P04394 | Thyrotropin-releasing hormone receptor signaling pathway | 2 | 5 | 10 | 7 | 2 | 6 | 4 | 2 | 9 |
| P05912 | Dopamine receptor mediated signaling pathway | 4 | 7 | 8 | 4 | 2 | 9 | 4 | 2 | 8 |
| P04398 | p53 pathway feedback loops 2 | 4 | 8 | 4 | 3 | 6 | 3 | 1 | 2 | 2 |
| P04377 | Beta1 adrenergic receptor signaling pathway | 2 | 5 | 7 | 3 | 0 | 4 | 2 | 1 | 5 |
| P05731 | GABA-B receptor II signaling | 1 | 7 | 3 | 3 | 1 | 3 | 2 | 2 | 4 |
| P06211 | BMP/activin signaling pathway-drosophila | 2 | 7 | 2 | 1 | 5 | 2 | 1 | 1 | 4 |
| P06212 | DPP-SCW signaling pathway | 2 | 7 | 2 | 1 | 5 | 2 | 1 | 1 | 4 |
| P06213 | DPP signaling pathway | 2 | 7 | 1 | 1 | 5 | 2 | 1 | 1 | 3 |
| P06216 | SCW signaling pathway | 2 | 7 | 2 | 1 | 5 | 2 | 1 | 1 | 4 |
| P04378 | Beta2 adrenergic receptor signaling pathway | 1 | 5 | 7 | 3 | 0 | 4 | 2 | 1 | 5 |
| P06587 | Nicotine pharmacodynamics pathway | 3 | 4 | 7 | 3 | 1 | 6 | 3 | 1 | 5 |
| P04373 | 5HT1 type receptor mediated signaling pathway | 1 | 5 | 4 | 3 | 1 | 6 | 3 | 1 | 5 |
| P06214 | GBB signaling pathway | 2 | 6 | 2 | 1 | 5 | 2 | 1 | 1 | 4 |
| P05918 | p38 MAPK pathway | 1 | 6 | 5 | 3 | 3 | 7 | 1 | 1 | 5 |
| P04397 | p53 pathway by glucose deprivation | 2 | 6 | 1 | 3 | 0 | 0 | 1 | 0 | 0 |
| P04385 | Histamine H1 receptor mediated signaling pathway | 1 | 4 | 6 | 5 | 2 | 1 | 2 | 0 | 5 |
| P04375 | 5HT3 type receptor mediated signaling pathway | 0 | 1 | 3 | 1 | 1 | 6 | 2 | 1 | 3 |
| P04376 | 5HT4 type receptor mediated signaling pathway | 0 | 2 | 4 | 3 | 1 | 6 | 3 | 1 | 4 |
| P05911 | Angiotensin II-stimulated signaling through G proteins and beta-arrestin | 1 | 2 | 5 | 3 | 2 | 1 | 1 | 0 | 4 |
| P05734 | Synaptic vesicle trafficking | 1 | 5 | 3 | 2 | 0 | 5 | 3 | 0 | 4 |
| P05913 | Enkephalin release | 1 | 5 | 2 | 2 | 0 | 0 | 1 | 0 | 3 |
| Note: Listed pathways involve at least one miR with five or more targets.  *: Pathway ID number of PANTHER | | | | | | |  |  |  |  |
